# Combined effect of genetic polymorphisms of *AURKA* and environmental factors on oral cancer development in Taiwan

**DOI:** 10.1371/journal.pone.0171583

**Published:** 2017-02-02

**Authors:** Chia-Hsuan Chou, Ying-Erh Chou, Chun-Yi Chuang, Shun-Fa Yang, Chiao-Wen Lin

**Affiliations:** 1 Institute of Medicine, Chung Shan Medical University, Taichung, Taiwan; 2 Department of Medical Research, Chung Shan Medical University Hospital, Taichung, Taiwan; 3 School of Medicine, Chung Shan Medical University, Taichung, Taiwan; 4 Department of Otolaryngology, Chung Shan Medical University Hospital, Taichung, Taiwan; 5 Institute of Oral Sciences, Chung Shan Medical University, Taichung, Taiwan; 6 Department of Dentistry, Chung Shan Medical University Hospital, Taichung, Taiwan; National Cancer Center, JAPAN

## Abstract

**Background:**

Oral squamous cell carcinoma (OSCC) is the sixth and fourth most common cause of cancer death in men worldwide and in Taiwan, respectively. *AURKA*, which encodes a centrosome-related serine/threonine kinase, is frequently amplified and overexpressed in many human cancers, particularly advanced OSCC. We conducted a hospital-based case-control study to estimate *AURKA* single-nucleotide polymorphisms (SNPs) and environmental risk factors to determine OSCC susceptibility and clinicopathological characteristics.

**Methodology/Principal findings:**

We enrolled a total of 876 OSCC patients and 1200 controls. Four SNPs of *AURKA*, namely rs1047972, rs2273535, rs2064863, and rs6024836, were analyzed using real-time polymerase chain reaction (PCR). Among the 1420 smokers, the *AURKA* polymorphism carriers with the betel nut chewing habit had a higher risk of oral cancer than *AURKA* wild-type (WT) carriers without the betel nut chewing habit. Patients with the *AURKA* rs2064863 gene had a 1.365-fold higher risk of stage III or IV OSCC (95% confidence interval [CI] 1.029–1.811) than those with the rs2064863 WT gene. Furthermore, carriers of the *AURKA* rs1047972/rs2273535/rs2064863 C-A-T haplotype had a 1.736-fold (95% CI 1.110–2.715) higher risk of OSCC than controls (C-T-T, the most common haplotype). Among patients with the betel quid chewing habit, carriers of other haplotypes (C-T-T, C-A-G, T-A-T, T-A-G, T-T-T, and C-T-G) had a 12.857-fold (95% CI 10.731–15.404) increased risk, and carriers of the C-A-T haplotype had the highest risk (AOR: 31.120; 95% CI 13.864–69.850) of OSCC, compared with those without the betel quid chewing who harbored other haplotypes.

**Conclusions:**

In conclusion, betel nut chewing combined with the *AURKA* C-A-T haplotypes lead to a high risk of OSCC. These findings reveal a novel genetic-environmental predisposition for oral tumorigenesis.

## Introduction

More than 90% of all head and neck malignant tumors occur in oral squamous cell carcinoma (OSCC) patients [1]. OSCC is the sixth and fourth most common cause of cancer death in men worldwide and in Taiwan, respectively [2]. Patients usually seek treatment only at the advanced stage of OSCC, resulting in a relatively low 5-year survival rate [3]. Both genetic factors and carcinogen-exposure behaviors (for example: betel nut chewing, alcohol consumption, and tobacco) regulate OSCC development [4, 5]. Moreover, our previous studies have demonstrated that genetic polymorphism combined with betel nut carcinogens may increase susceptibility to OSCC [6–12]. The results illustrate the importance of single-nucleotide polymorphisms (SNPs) for predicting risk or prognosis of OSCC.

*AURKA*, also known as *Aurora Kinase A*, encodes a centrosome-related serine/threonine kinase and is frequently amplified and overexpressed in many human cancers [13–15], particularly advanced OSCC [16]. Moreover, this gene has been identified as a definite low-penetrance tumor susceptibility gene [17]. The high expression of *AURKA* might be induced by centrosome amplification, aberrant chromosome segregation, aneuploidy, and malignant transformation [18–20], thus mediating the molecular mechanisms underlying carcinogenesis. The genetic associations of *AURKA* with several conditions have been documented. Lee et al demonstrated that the AA genotype of AURKA rs2273535 T>A was associated with an increased risk of oral cancer [21]. Dai et al reported that Caucasians harboring AURKA rs1047972 T>C had a reduced breast cancer risk [22]. However, few genetic variants of AURKA have been associated with OSCC.

In this case-control study, we investigated the relationship of four *AURKA* polymorphisms—namely rs1047972, rs2273535, rs2064863, and rs6024836—with OSCC susceptibility in Taiwanese male patients with OSCC.

## Results

### Patient characteristics and distribution of oral cancer

The distributions of the demographic characteristics of the study subjects are summarized in Table 1. A total of 876 male patients with oral cancer and 1200 male controls were included in this study. The mean age ± SD in the controls and patients was 53.90 ± 10.02 and 54.80 ± 11.03 years, respectively. A significant difference was observed in the prevalence of betel nut chewing, cigarette smoking, and alcohol drinking between oral cancer patients and controls.

**Table 1:** The distributions of demographical characteristics in 1200 controls and 876 male patients with oral cancer.

Variable	Controls (N = 1200)	Patients (N = 876)	p value
Age (yrs)			
	53.90 ± 10.02	54.80 ± 11.03	p = 0.06
Betel quid chewing			
No	1001 (83.4%)	175 (20.0%)	
Yes	199 (16.6%)	701 (80.0%)	p <0.01*
Cigarette smoking			
No	564 (47.0%)	92 (10.5%)	
Yes	636 (53.0%)	784 (89.5%)	p <0.01*
Alcohol drinking			
No	963 (80.3%)	388 (44.3%)	
Yes	237 (19.8%)	488 (55.7%)	p <0.01*
Stage			
I+II		435 (49.7%)	
III+IV		441 (50.3%)	
Tumor T status			
T1+T2		512 (58.4%)	
T3+T4		364 (41.6%)	
Lymph node status			
N0		597 (68.2%)	
N1+N2+N3		279 (31.8%)	
Metastasis			
M0		868 (99.1%)	
M1		8 (0.9%)	
Cell differentiation			
Well differentiated		141 (16.1%)	
Moderately or poorly differentiated		735 (83.9%)	

Mann-Whitney U test or Fisher’s exact test was used between healthy controls and patients with oral cancer.

* p value < 0.05 as statistically significant.

### Associations between *AURKA* SNP and oral cancer

In the control group, the genotypic frequencies of *AURKA* SNP rs1047972 C/T, rs2273535 A/T, rs2064863, and rs6024836 A/G were in Hardy-Weinberg equilibrium (*p >* 0.05). The genotypic and allelic frequencies of *AURKA* SNPs in oral cancer patients and controls are shown in Table 2. After adjustment for age, betel quid chewing, cigarette smoking, and alcohol drinking, no significant difference was observed between oral cancer patients and controls.

**Table 2:** Genotyping and allele frequency of *AURKA* single nucleotide polymorphism in oral cancer and normal controls.

Variable	Controls N = 1200(%)	Patients N = 876 (%)	AOR (95% CI)	p value
**rs1047972**				
CC	925 (77.1%)	668 (76.3%)	1.00	
TC	261 (21.8%)	190 (21.7%)	0.93 (0.70–1.23)	p = 0.60
TT	14 (1.1%)	18 (2.0%)	1.95 (0.74–5.13)	p = 0.18
TC+TT	275 (22.9%)	208 (23.7%)	0.97 (0.74–1.27)	p = 0.84
C allele	2111 (88.0%)	1526 (87.1%)	1.00	
T allele	289 (12.0%)	226 (12.9%)	1.02 (0.80–1.31)	p = 0.87
**rs2273535**				
TT	583 (48.6%)	404 (46.1%)	1.00	
AT	490 (40.8%)	368 (42.0%)	1.16 (0.91–1.48)	p = 0.25
AA	127 (10.6%)	104 (11.9%)	1.03 (0.70–1.51)	p = 0.88
AT+AA	617 (51.4%)	472 (53.9%)	1.13 (0.90–1.42)	p = 0.31
T allele	1656 (69.0%)	1176 (67.1%)	1.00	
A allele	774 (31.0%)	576 (32.9%)	1.06 (0.89–1.26)	p = 0.50
**rs2064863**				
TT	827 (68.9%)	590 (67.4%)	1.00	
GT	338 (28.2%)	252 (28.8%)	1.01 (0.79–1.31)	p = 0.92
GG	35 (2.9%)	34 (3.8%)	1.21 (0.64–2.30)	p = 0.55
GT+GG	373 (31.1%)	286 (32.6%)	1.03 (0.81–1.32)	p = 0.80
T allele	1992 (83.0%)	1432 (81.7%)	1.00	
G allele	408 (17.0%)	320 (18.3%)	1.05(0.85–1.29)	p = 0.67
**rs6024836**				
AA	525 (43.8%)	352 (40.2%)	1.00	
AG	530 (44.2%)	397 (45.3%)	1.15 (0.90–1.47)	p = 0.26
GG	145 (12.0%)	127 (14.5%)	1.19(0.83–1.70)	p = 0.36
AG+GG	675 (56.2%)	524 (59.8%)	0.86 (0.68–1.09)	p = 0.22
A allele	1580 (65.8%)	1101 (62.8%)	1.00	
G allele	820 (34.2%)	651 (37.2%)	1.11 (0.94–1.31)	p = 0.24

In Table 3, among the 1420 smokers, the subjects with *AURKA* rs1047972, rs2273535, rs2064863, and rs6024836 polymorphisms who exhibited the betel nut chewing habit respectively had 10.589-fold (95% confidence interval [CI] 6.994–16.032), 12.663-fold (95% CI 8.633–18.575), 17.912-fold (95% CI 6.596–48.643), and 13.912-fold (95% CI 9.392–20.607) significantly higher risks of OSCC than did smokers with wild-type genes without the betel nut chewing habit.

**Table 3:** Associations of the combined effect of *AURKA* gene polymorphisms and betel nut chewing with the susceptibility to oral cancer among 1420 smokers.

Variable	Controls (n = 636) (%)	Patients (n = 784) (%)	OR (95% CI)	AOR (95% CI)
**rs1047972**				
CC genotype & non-betel nut chewing	348 (54.7%)	93 (11.9%)	1.00	1.00
TC or TT genotype or betel nut chewing	244 (38.4%)	535 (68.2%)	**8.21 (5.87–11.47)**	**6.80 (4.82–9.59)**
TC or TT genotype with betel nut chewing	44 (6.9%)	156 (19.9%)	**13.27 (8.09–21.77)**	**10.59 (6.38–17.57)**
**rs2273535**				
TT genotype & non-betel nut chewing	226 (35.5%)	53 (6.8%)	1.00	1.00
AT or AA genotype or betel nut chewing	313 (49.2%)	376 (48.0%)	**5.12 (3.40–7.71)**	**4.28 (2.81–6.51)**
AT or AA genotype with betel nut chewing	97 (15.3%)	355 (45.2%)	**15.61 (9.88–24.64)**	**12.66 (7.93–20.22)**
**rs2064863**				
TT genotype & non-betel nut chewing	318 (50.0%)	80 (10.2%)	1.00	1.00
GT or GG genotype or betel nut chewing	313 (49.2%)	676 (86.2%)	**8.59 (6.10–12.08)**	**7.25 (5.11–10.29)**
GT or GG genotype with betel nut chewing	5 (0.8%)	28 (3.6%)	**22.25 (6.70–73.89)**	**17.91 (5.29–60.69)**
**rs6024836**				
AA genotype & non-betel nut chewing	213 (33.5%)	46 (5.9%)	1.00	1.00
AG or GG genotype or betel nut chewing	314 (49.4%)	342 (43.6%)	**5.04 (3.27–7.77)**	**4.21 (2.70–6.58)**
AG or GG genotype with betel nut chewing	109 (17.1%)	396 (50.5%)	**16.82 (10.54–26.85)**	**13.91 (8.61–22.48)**

The adjusted odds ratios (AORs) with their 95% confidence intervals (CIs) were estimated by multiple logistic regression models after controlling for age and alcohol drinking.

### Associations between *AURKA* SNPs and the clinicopathologic status of OSCC

We further clarified the role of *AURKA* polymorphisms in the clinicopathologic status of OSCC, such as the tumor clinical stage, tumor size, lymph node metastasis, and cell differentiation. Among the 876 oral cancer patients, only patients with the *AURKA* rs2064863 gene had a 1.365-fold higher risk of stage III or IV OSCC (95% CI 1.029–1.811) than did patients with the rs2064863 wild-type gene (p = 0.031). However, no significant difference was observed in the tumor size, lymph node metastasis clinical stage, lymph node metastasis, or cell differentiation (Table 4).

**Table 4:** Effect of *AURKA* rs2064863 polymorphism on clinical statuses in 786 male oral cancer.

	*AURKA* rs2064863			
Variable	TT (n = 590) n (%)	GT+GG (n = 286) n (%)	OR (95% CI)	AOR (95% CI)^a^
**Clinical Stage**				
Stage I/II	278 (47.1%)	157 (54.9%)	1.00	1.00
Stage III/IV	312 (52.9%)	129 (45.1%)	**1.37 (1.04–1.82)**^b^	**1.37 (1.03–1.81)**^c^
**Tumor size**				
≤T2	333 (56.4%)	179 (62.6%)	1.00	1.00
> T2	257 (43.6%)	107 (37.4%)	0.78 (0.58–1.04)	0.78 (0.58–1.04)
**Lymph node metastasis**				
No	392 (66.4%)	205 (71.7%)	1.00	1.00
Yes	198 (33.6%)	81 (28.3%)	0.77 (0.57–1.05)	0.78 (0.57–1.06)
**Cell differentiation**				
well	97 (16.4%)	44 (15.4%)	1.00	1.00
Moderate/poor	493 (83.6%)	242 (84.6%)	2.70 (0.709–10.30)	2.68 (0.70–10.24)

^a^ Adjusting for the effects of age, betel quid chewing, cigarette smoking and alcohol drinking

^b^ p = 0.03

^c^ p = 0.03

### Haplotype analysis of the *AURKA* gene

We used Haploview software and the PHASE program to calculate pairwise linkage disequilibrium (LD) and analyzed the common haplotypes. As shown in Table 5, the p value for the global test of five haplotypes was 0.002 for OSCC development. The most common haplotype was C-T-T (68.4%) in the control group; thus, this haplotype was used as the haplotype reference. Compared with the reference group, carriers with C-A-T or other haplotypes had 1.736-fold (95% CI 1.110–2.715) and 2.788-fold (95% CI 1.387–5.605) significantly increased risks of OSCC (Table 5). Furthermore, we estimated the combined effect of betel quid chewing and *AURKA* haplotypes on OSCC development (Table 6). Patients with other haplotypes (C-T-T, C-A-G, T-A-T, T-A-G, T-T-T, and C-T-G) without the betel quid chewing habit were selected as a reference group. After adjustment for the effects of age, cigarette smoking, and alcohol drinking, patients with the betel quid chewing habit and other haplotypes had a 12.857-fold (95% CI 10.731–15.404) (p<0.001) increased risk. Moreover, those with the betel quid chewing habit and the C-A-T haplotype had the highest risk of OSCC (AOR 31.120; 95% CI 13.864–69.850; p<0.001).

**Table 5:** Frequencies of *AURKA* haplotypes in OSCC patients and control subjects.

Haplotype block			Controls n = 2400	Patients n = 1752	OR (95% CI)	AOR (95% CI)^a^
rs1047972 T/C	rs2273535 A/T	rs2064863 T/G				
Global haplotype test (p = 0.0018)						
C	T	T	1641 (68.4%)	1170 (66.8%)	1.00	1.00
C	A	G	399 (16.6%)	282 (16.1%)	0.99 (0.84–1.18)	0.95 (0.76–1.18)
T	A	T	269 (11.2%)	190 (10.8%)	0.99 (0.81–1.21)	0.92 (0.71–1.20)
C	A	T	69 (2.9%)	70 (4.0%)	1.42 (1.01–2.00)^c^	1.74 (1.11–2.72)^e^
Others^b^			22 (0.9%)	40 (2.2%)	2.55 (1.51–4.31)^d^	2.79 (1.39–5.61)^f^

^a^ Adjusting for the effects of age, betel quid chewing, cigarette smoking and alcohol drinking

^b^ Others: TAG (41), TTT (15), and CTG (6)

^c^ p = 0.04

^d^ p < 0.01

^e^ p = 0.02

^f^ p < 0.01

**Table 6:** Combined effect of betel quid chewing and *AURKA* haplotypes on OSCC development.

Betel quid chewing	*AURKA* haplotype	Controls n = 2400	Patients n = 1752	AOR (95% CI)^b^
Yes	C-A-T	7 (0.3%)	55 (3.1%)	31.09 (13.86–69.79)^c^
Yes	Others^a^	391 (16.3%)	1347 (76.9%)	12.81 (10.70–15.35)^c^
No	C-A-T	62 (2.6%)	15 (0.9%)	1.44 (0.82–2.67)
No	Others^a^	1940 (80.8%)	335 (19.1%)	1.00

^a^ Other haplotypes included C-T-T, C-A-G, T-A-T, T-A-G, T-T-T, and C-T-G

^b^ Adjusting for the effects of age, cigarette smoking and alcohol drinking

^c^ p < 0.01

## Discussion

In this study, we observed that the combination of the *AURKA* gene polymorphisms with betel nut chewing increased OSCC susceptibility. Furthermore, we observed an interaction between the clinicopathological statuses and *AURKA* rs2064863 polymorphism. The results of pairwise allele analysis for rs1047972/rs2273535/rs2064863 revealed that the C-A-T haplotype is associated with the risk of OSCC.

AURKA overexpression has been demonstrated in various human cancers, including breast cancer, head and neck squamous cell carcinoma, colorectal cancer, ovarian cancer, and advanced OSCC [13–16, 23]. The abnormal expression of AURKA might lead to high chromosome instability in tumors and further increase susceptibility to malignant transformation [24]. These processes may result from the gaining of the chromosome 20q amplicon, which promotes the progression of adenoma to carcinoma [25]. Activation of the Wnt/β-catenin and PI3K/Akt signaling pathways plays a crucial role in cancer development [26]. Moreover, AURKA overexpression promotes cell proliferation and tumor progression and metastasis [27–29]. Furthermore, the blocking of AURKA induces autophagy and apoptosis in mice and significantly increases sensitivity to chemical treatment in OSCC [16, 30]. Taken together, these findings indicate that AURKA knockdown might be a valuable therapeutic strategy for OSCC.

A previous study demonstrated that the *AURKA* 91A (rs2273535) allele polymorphism is associated with a high risk of oral cancer [21]. Previous studies have also suggested that the *AURKA* rs2273535 polymorphism is associated with a high risk of breast cancer, particularly in Asians [31]. However, the *AURKA* rs1047972 polymorphism has been shown to reduce the incidence of breast cancer in Caucasians [22]. In this study, our data showed that *AURKA* polymorphisms were not associated with the risk of OSCC (Table 2). However, the significant interaction between the investigated *AURKA* polymorphisms and betel nut chewing was associated with a high incidence of OSCC in smokers. These results suggest that environmental risk factors such as betel nut chewing and tobacco smoking influence AURKA gene expression, subsequently leading to a high incidence of OSCC.

The present study demonstrated that the *AURKA* rs2064863 polymorphism was associated with a high risk of stage III/IV OSCC but not with tumor size, metastasis to the lymph node and distant organs, or cell differentiation (Table 4). Chuang et al reported that AURKA gene overexpression was strongly associated with the progression of colorectal adenoma to colorectal cancer. AURKA overexpression may cause high chromosome instability in tumors and further increase susceptibility to malignant transformation [24]. Sillard-Hardebol et al also observed the same results [25]. However, the possibility of an association among the advanced stage, AURKA expression level, and *AURKA* genotype, as well as the effects of the *AURKA* genotype on oral cancer risk, require further investigation.

A study found that the *AURKA* rs2273535 polymorphism increases the risk of breast cancer, and that the rs1047972 polymorphism is a protective factor for breast cancer [22]. A study conducted in Korea revealed that the *AURKA* rs2273535 polymorphism was in strong LD with the rs1047972 genotype, and that patients with the *AURKA* haplotype variants had high kinase activity and a high risk of progression to advanced stage gastric cancer [32]. In the present study, we observed that subjects with the rs1047972/rs2273535/rs2064863 C-A-T haplotype had a higher risk of OSCC than those with the common C-T-T haplotype. These results indicate that the *AURKA* gene variants might have different functional roles in different cancers.

The incidence of OSCC is particularly high in Taiwan because of the popularity of betel nut chewing. Approximately 2.5 million people in Taiwan have the betel nut chewing habit [33]. Several studies have indicated that betel quid may damage the oral mucosa, inducing genotoxic or nongenotoxic effects, which are associated with the initiation, promotion, and progression of OSCC [34–39]. In our study, betel nut chewing was related to OSCC development. Therefore, we estimated the combined effect of betel nut chewing and *AURKA* haplotypes on OSCC development. Among patients without the betel nut chewing habit, those with the *AURKA* rs1047972/rs2273535/rs2064863 C-A-T haplotype had a 1.443-fold (95% CI 0.801–2.601) higher risk of OSCC than those with other haplotypes, but the risk was nonsignificant. Moreover, we observed that betel nut users with other *AURKA* haplotypes had an increased risk of OSCC. Thus, the increased risk might result from betel nut chewing. Betel nut users with the *AURKA* rs1047972/rs2273535/rs2064863 C-A-T haplotype had a higher risk of OSCC. Previous studies have demonstrated that *AURKA* 91A (Ile31) is a low-penetrance tumor susceptibility allele, and individuals homozygous or heterozygous for this allele tend to exhibit an increased risk of several human cancers [40–45]. Matarasso et al confirmed that the expression level of *AURKA* mRNA was higher in Ile31/Ile31-homozygous (2.07-fold) and Phe31/Ile31-heterozygous (1.93-fold) samples from normal prostate tissues than in Phe31/Phe31-homozygous samples [46]. In addition, the *AURKA* rs1047972 polymorphism, which results in a valine-to-isoleucine substitution, has been investigated for its association with breast cancer, and this polymorphism has been found to be a protective factor for breast cancer in Caucasians but not in Asians [22, 47]. The effect of this SNP on OSCC has not been clarified. Betel nut chewing may induce OSCC, and betel quid components can stimulate the activation of Src and ERK for promoting the migration and motility of cancer cells [48]. Thus, betel nut chewing might alter AURKA expression, thereby impairing cell growth, differentiation, and inflammation.

Our study has some limitations. First, our data were collected from two medical centers and may have referral bias; future studies can therefore collect data from different regions in Taiwan to verify our present data. Second, the questionnaire on betel nut, tobacco, and alcohol use described users as “ever users” and “never users.” Data on the amount, length, and history of betel nut, tobacco, and alcohol use could not be collected to conduct a comprehensive analysis.

In conclusion, betel nut users with the *AURKA* C-A-T haplotypes have a high risk of OSCC. In addition, the SNP rs2064863 is associated with a high risk of developing advanced-stage tumors. These findings reveal a novel genetic-environmental predisposition for oral tumorigenesis.

## Materials and methods

### Study subjects and specimen collection

In this study, we enrolled 876 male patients with OSCC from Chung Shan Medical University Hospital in Taichung, Taiwan from 2007 to 2015. In the 876 patients, the tumors were located in the buccal mucosa, tongue, gingiva, palate, floor of the mouth, and other sites. For the control group, we selected 1200 healthy male individuals with no self-reported history of cancer at any site from the Taiwan Biobank. Each subject completed a questionnaire on their demographic characteristics, betel nut chewing, tobacco use, and alcohol consumption, and medical history. Whole blood specimens collected from OSCC patients and controls were placed in tubes containing ethylenediamine tetra-acetic acid (EDTA), immediately centrifuged, and stored at −80°C. All participants were provided with a written description of the study. The Institutional Review Board of Chung Shan Medical University Hospital approved this study (CSMUH No: CS13214-1), and informed written consent was obtained from each participant.

### Determination of genotypes

Genomic DNA was isolated from peripheral blood using QIAamp DNA blood mini kits (Qiagen, Valencia, CA, USA) following manufacturer instructions. DNA was dissolved in TE buffer (10 mM Tris and 1 mM EDTA acid; pH 7.8) and quantified by measuring the optical density at 260 nm. The final preparation was stored at −20°C and used as templates for polymerase chain reaction (PCR). Allelic discrimination of the rs1047972, rs2273535, rs2064836, and rs6024836 AURKA polymorphisms was performed using TaqMan SNP genotyping assays with the ABI StepOne^™^ Real-Time PCR System (Applied Biosystems, Foster City, CA, USA), and these polymorphisms were further analyzed using SDS version 3.0 (Applied Biosystems).

### Statistical analysis

Hardy-Weinberg equilibrium was assessed using a chi-square goodness-of-fit test for bi-allelic markers. The Mann-Whitney U test and Fisher’s exact test were used to compare differences in the distribution of age and demographic characteristics between the controls and OSCC patients. ORs with 95% CIs were estimated using logistic regression models. AORs with 95% CIs were used to assess association between genotype frequencies with OSCC risk and clinical factors. Moreover, Bonferroni correction is used to adjust for multiple comparisons. p values less than 0.05 were considered significant. The data were analysed with SPSS 12.0 statistical software (SPSS Inc., Chicago, IL, USA).
